# Editorial Note: The ‘side effects’ of digitalization: A study on role overload and job burnout of employees

**DOI:** 10.1371/journal.pone.0359366

**Published:** 2026-09-29

**Authors:** The PLOS One Editors

The *PLOS One* Editors issue this Editorial Note to inform readers that work cited in this article [1] as Reference 23 was retracted after the article’s publication date. PLOS considers that this reference is not crucial in supporting the research or conclusions reported in [1].

In addition, the *PLOS One* Editors have concerns about the article’s [1] compliance with the PLOS Authorship policy.

The corresponding author stated that all authors satisfy the requirements set out in the PLOS Authorship policy. The Editors do not consider this matter resolved.
